# Association between delayed initiation of adjuvant CMF or anthracycline-based chemotherapy and survival in breast cancer: a systematic review and meta-analysis

**DOI:** 10.1186/1471-2407-13-240

**Published:** 2013-05-16

**Authors:** Ke-Da Yu, Sheng Huang, Jia-Xin Zhang, Guang-Yu Liu, Zhi-Ming Shao

**Affiliations:** 1 Department of Breast Surgery, Cancer Center and Cancer Institute, Shanghai Medical College, Fudan University, 399 Ling-Ling Road, Shanghai 200032, People’s Republic of China

## Abstract

**Background:**

Adjuvant chemotherapy (AC) improves survival among patients with operable breast cancer. However, the effect of delay in AC initiation on survival is unclear. We performed a systematic review and meta-analysis to determine the relationship between time to AC and survival outcomes.

**Methods:**

PubMed, EMBASE, Cochrane Database of Systematic Reviews, and Web-of-Science databases (between January-1 1978 and January-29, 2013) were searched for eligible studies. Hazard ratios (HRs) for overall survival (OS) and disease-free survival (DFS) from each study were converted to a regression coefficient (β) corresponding to a continuous representation per 4-week delay of AC. Most used regimens of chemotherapy in included studies were CMF (cyclophosphamide, methotrexate, and fluorouracil) or anthracycline-based. Individual adjusted β were combined using a fixed-effects or random-effects model depending on heterogeneity.

**Results:**

We included 7 eligible studies with 9 independent analytical groups involving 34,097 patients, 1 prospective observational study, 2 secondary analyses in randomized trials (4 analytical groups), and 4 hospital-/population-based retrospective study. The overall meta-analysis demonstrated that a 4-week increase in time to AC was associated with a significant decrease in both OS (HR = 1.15; 95% confidence interval [CI], 1.03-1.28; random-effects model) and DFS (HR = 1.16; 95% CI, 1.01-1.33; fixed-effects model). One study caused a significant between-study heterogeneity for OS (P < 0.001; I^2^ = 75.4%); after excluding that single study, there was no heterogeneity (P = 0.257; I^2^ = 23.6%) and the HR was more significant (HR = 1.17; 95% CI, 1.12-1.22; fixed-effects model). Each single study did not fundamentally influence the positive outcome and no evidence of publication bias was observed in OS.

**Conclusions:**

Longer time to AC is probably associated with worse survival in breast cancer patients.

## Background

Breast cancer is one of the most common cancers in women in both developed and developing countries. Several large clinical trials and meta-analysis of all the relevant randomized trials of adjuvant systemic therapy have consistently demonstrated that chemotherapy decreases 30-40% risk of breast cancer mortality versus those without chemotherapy [1]. Adjuvant chemotherapy (AC) is routinely recommended to most of breast cancer patients post surgeries. National Comprehensive Cancer Network guidelines (http://www.nccn.org) recommend patients with tumor larger than 1 cm or having involved nodes to receive AC; while St. Gallen consensus recommends patients with endocrine non- or less-responsive disease to undergo AC [2]. Clinically, 60-80% of breast cancer patients would ultimately receive AC, but the optimal time from surgery to the start of chemotherapy is unclear albeit clinicians have used chemotherapy in breast cancer for more than a half century. Oncologists might suggest start of AC within 6 to 8 weeks after surgery based on a routine clinical assumption that AC should commence as soon as practical. Some clinicians might also harbor the assumption that chemotherapy would have little or no adjuvant benefit beyond a delay of 3 months [3]. However, there is no direct evidence supporting either of these assumptions. Of note, in practice, not all patients could initiate AC in this time frame, and some have to face a delay in AC due to postoperative complications, personal decision of receiving AC, comorbid conditions, or health-system logistic factors such as delays in referral or waiting times.

Time window of AC treatment remains an important issue. Regrettably, this issue has not been subjected to a randomized controlled clinical trial; nor is such trial likely to be undertaken due to its low operability, poor patient compliance, and potential ethical problems. Several retrospective studies [4-7], observational prospective studies [8], and retrospective analyses on clinical trial data [9,10], have examined the impact of early and delayed initiation of AC on survival of breast cancer patients, but the results are inconsistent. To address this important gap, we undertook a systematic review of all the relevant literatures and performed a quantitative meta-analysis to assess the relationship between a delay in AC and survival in breast cancer.

## Methods

### Literature search

The literature search was conducted in the PubMed, EMBASE, Cochrane Database of Systematic Reviews, and Web-of-Science databases (between January-1 1978 and January-29 2013). Potentially relevant studies were identified using following keywords: “(Timing or time) and adjuvant and (chemotherapy or chemotherapeutic) and breast cancer and survival”. The reference lists from relevant papers, especially from review articles, were checked to identify more studies unidentified in the original search. Online available abstracts of the annual meetings of the American Society of Clinical Oncology (2007–2011) were searched for newly completed studies. This systematic review and meta-analysis was planned, conducted, and reported in adherence to the standards of quality for reporting meta-analysis [11]. The basic procedure of meta-analysis was performed as described previously [12-14].

### Eligibility and validity of literature-based data

The citations from the initial search were subsequently screened for eligibility. Studies included in the systematic review and meta-analysis should meet the following criteria: (1) All patients with operable primary breast cancer were treated with AC, with documented time from surgery to initiation of AC. (2) The relationship between time interval from surgery to AC and patient outcomes in breast cancers was reported. The outcomes could be presented as disease-free survival (DFS), event-free survival (EFS), relapse-free survival (RFS), or overall survival (OS). Hazard ratio (HR) with 95% confidence intervals (CIs) (or sufficient data to calculate them) was reported. (3) To minimize the effect of confounding between comparison groups, only studies identified as “high validity” by the following criteria were included in the pooling analysis: first, the relevant prognostic factors were adequately described between comparator groups; second, either the comparison groups were balanced for the relevant prognostic factors, or the reported results were adjusted for other prognostic factors [13]. (4) Studies that used nonstandard forms of AC (e.g., perioperative, dose-dense, or neoadjuvant chemotherapy), or examined the effect of concurrent or sequencing of additional adjuvant therapies (e.g., endocrine therapy or radiotherapy) were excluded. (5) To reduce the effect of publication bias, all publish types either full-text article, correspondence, or meeting abstract were eligible. But studies should be published in English. Three reviewers (Y.K.D., H.S., and S.Z.M.) independently assessed studies for inclusion with disagreements resolved by consensus. The study quality was assessed using the 9-star Newcastle-Ottawa Scale (The Newcastle-Ottawa Scale for assessing the quality of nonrandomized studies in meta-analyses. Ottawa, Canada: Dept of Epidemiology and Community Medicine, University of Ottawa. http://www.ohri.ca/programs/clinical_epidemiology/oxford.htm. Accessible on March-1, 2013).

### Estimating HR for adverse outcomes per 4-week delay in AC

This step was mainly performed according to the procedure described previously with a few modifications [13,14]. Briefly, the measure of effect in all studies was a HR for OS and/or DFS. In most studies, EFS or RFS had the same or similar definition to DFS and thus was treated as DFS when appropriate. The eligible studies used disparate categorical representations of waiting time. To provide a common representation for synthesis of the results of individual studies, we converted the waiting time effect size to a regression coefficient (β) and its standard error (SE) corresponding to a continuous representation per 4-week of delay. For the waiting time categories in each article, a central value was assigned to each category. For studies with 2 waiting time groups, since the authors defined the 2 groups as “before *n* weeks (not delayed AC)” and “after *n* weeks (delayed AC)”, we treated the reference time level as *n*/2 weeks and the exposure time level as *n*/2 + *n* weeks. the weekly β was calculated as ln(HR)/(X*n* - X0), and the corresponding SE of β was calculated as (ln[upper of 95% CI]-ln[lower of 95% CI])/([X*n* - X0]*1.96*2), where CI is confidence interval, X*n* denotes exposure at N level by time (week), and X0 denotes exposure at reference time level. We transferred all time unit (day, week, or month) to “week” and “N” in the X*n* was assigned to the number of week. The value of 1.96 might change according to the significance level in each study. If only a P-value was provided, the SE was calculated as the “test-based” method: SE of ln(HR) = (ln[HR])/Z_p_, where Z_p_ is the value of a unit-normal test (e.g., Z_p_ = 1.96 when P = 0.05, 2-sided test). For the studies with more than 2 categories, the weighted least-squares linear regression of the ln(HR) on every exposure level in one study was used to estimate the summary β as previously described [15,16]. The dependent variable for the regression was the log of each study-specific HR, weighted by the inverse of its variance. The summary measures of HR per 4-week of delay from each study presented here can be interpreted as the incidence rate ratio for the outcome with each 4-week of additional waiting for AC, which could be calculated by *e*^β*4^. We made all the above calculations assuming a log linear relationship between HR and delayed time.

### Meta-analysis

The adjusted regression coefficients from individual studies were combined using a fixed-effects or random-effects model according to absence or presence of between-study heterogeneity, respectively. Q statistic and I^2^ were used to evaluate the statistical heterogeneity between studies [17]. Heterogeneity was considered as either a P-value <0.05 or I^2^ >25% [18]. The inverse variance was used to weight individual studies. We performed influence analysis (sensitivity analysis) by omitting each study to find the potential outliers. The potential publication bias was examined visually in a funnel plot of log(HR) against its SE, and the degree of asymmetry was tested using Egger’s test [19] (P < 0.05 considered to be statistically significant). All of the statistical analysis was performed using Stata v.10.0 (Stata Corporation, College Station, TX) and SPSS 17.0 (SPSS Inc, Chicago, IL). Two-sided P < 0.05 was considered statistically significant.

## Results

**Figure 1:**
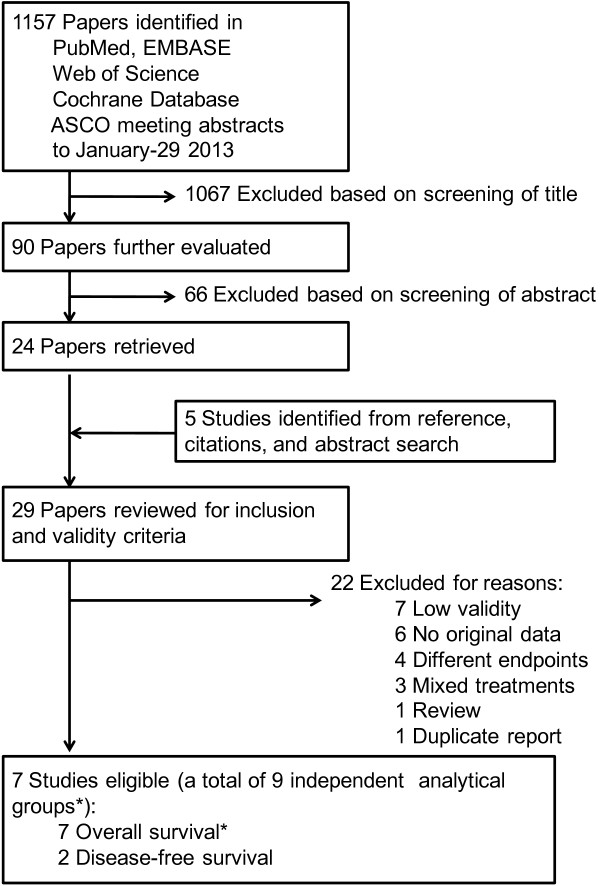
**The literature search process.** Validity required that either the comparison groups were balanced for relevant prognostic factors or the reported results were adjusted for these prognostic factors (Refer to the “Methods” section). *One study includes 3 analytical groups in overall survival.

The flow diagram of literature search is shown in Figure 1. The search strategy yielded 1,157 reports, of which 29 were potentially eligible after reviewing their abstracts. Twenty-one items were further excluded either because of a lack of data or because they did not meet the high validity criteria, leaving 7 eligible papers including 7 independent analytical groups for OS and 2 for DFS, respectively (Table 1). The studies were published between 1989 and 2013. There were 34,097 patients with primary breast cancer, with a range of sample size from 229 to 14,380. Two studies (4 analytical groups) reported time to AC data as a secondary analysis within randomized controlled trials of chemotherapy treatment [9], 1 study was conducted prospectively [8,10], and the left 4 were retrospective investigations using hospital- or population-based data [5-7,20].

**Table 1:** Characteristics of eligible studies on waiting time to adjuvant chemotherapy and survival in breast cancer

**Source**	**Place, data type and name**	**Median age, year**	**Menopausal status**	**Stage**	**Hormone receptor-positive (%)**	**Chemotherapy**	**Median FU**	**WT categories**	**Sample size**	**Additional survival data**	Study quality**	**Adjustment for covariates**
							**Outcome and HR (95% CI)**					
Pronzato et al [8] 1989	Italy (Pros.)	51 yr (range, 27–70)	Mixed	Operable (LN+)	NR	CMF	Median FU: 37 months	Total	229	4-yr OS:78%	7	Age, nodes status, menopausal status, cycle number, individual dose intensity
							Reference	≤35 days	116	4-yr OS:88%		
							OS, 2.61 (1.26-5.39)	>35 days	113	4-yr OS:69%		
Colleoni et al. [9] 2000	Multicenter (CT, IBCSG)	78% pts ≥40 yr	Pre.	Operable (LN+)	66.2	CMF	Median FU: 7.7 years	Total	1,788		8	Age, size, nodal status, vessel invasion, and institution
							DFS, 0.88 (0.76-1.03)	<21 days	599	5-yr DFS 62%; 10-yr DFS 51%		
							Reference	≥21 days	1,189	5-yr DFS 57%; 10-yr DFS 42%		
Kerbrat, et al. [5] 2005^*^	France (Retros., FASG)	NR	NR	Operable	NR	Anthr.-based	Median FU: 9 years	Total	2,602		7	Multivariate adjustment; adjusted factors not reported
							DFS, 0.85 (0.65-1.05)^§^	< 28 days	1,614	9-yr DFS 60%		
							Reference	28-42 days	883	9-yr DFS 58%		
								>42 days	105	9-yr DFS 49%		
Cold et al. [10] 2005 (I)	Denmark (CT, DBCG)	53% pts <46 yr	Mixed	Operable	77.0	Classical CMF	Median FU: NR	Total	352		6	Age, tumour size, nodes status, histological type, grade, hormone receptor status, and adjuvant irradiation
		43% pts 46–55 yr					Reference	1-3 wks	58			
		3% pts >55 yr					OS, 0.929 (0.441-1.957)	3-4 wks	92			
							OS, 1.549 (0.761-3.149)	4-5 wks	75			
							OS, 1.588 (0.856-2.948)	5-13 wks	127			
Cold et al. [10] 2005 (II)	Denmark (CT, DBCG)	40% pts <46 yr 40% pts 46–55 yr 20% pts >55 yr	Mixed	Operable	58.3	CMF i.v.	Median FU: NR	Total	6,065		8	Age, tumour size, nodes status, histological type, grade, hormone receptor status, and adjuvant irradiation
							Reference	1-3 wks	1,509			
							OS, 1.021 (0.903-1.155)	3-4 wks	1,581			
							OS, 0.890 (0.782-1.012)	4-5 wks	1,423			
							OS, 1.002 (0.884-1.136)	5-13 wks	1,552			
Cold et al. [10] 2005 (III)	Denmark (CT, DBCG)	47% pts <46 yr 41% pts 46–55 yr 12% pts >55 yr	Mixed	Operable	61.8	CEF	Median FU: NR	Total	1,084		7	Age, tumour size, nodes status, histological type, grade, hormone receptor status, and adjuvant irradiation
							Reference	1-3 wks	188			
							OS, 1.218 (0.800-1.854)	3-4 wks	305			
							OS, 1.045 (0.716-1.525)	4-5 wks	263			
							OS, 1.238 (0.861-1.782)	5-13 wks	328			
Hershman et al. [6] 2006	USA (Retros., SEER)	100% pts ≥65 yr	Post.	I-II	67.6	Polychemotherapy	Median FU: NR	Total	5,003		8	Age, race, live location, stage, hormone receptor, grade, comorbid conditions, SES score, marital status, teaching hospital, surgery, and radiation
							Reference	<1 month	2,361			
							OS, 1.00 (0.88-1.14)	1-2 months	1,846			
							OS, 1.08 (0.85-1.36)	2-3 months	323			
							OS, 1.46 (1.21-1.75)	>3 months	477			
Lohrisch et al. [7] 2006	USA (Retros.,)	47 yr	Mixed	I-II	60.0	CMF and Anthr.-based	Median FU: 6.2 years	Total	2,594		8	Age, size, nodal status, lymphatic or vascular invasion, and anthracycline
							Reference	≤4 wks	993	5-yr EFS 72.7%; 5-yr OS 83.5%		
								4-8 wks	1,272	5-yr EFS 77.3%; 5-yr OS 85.1%		
								8-12 wks	217	5-yr EFS 82.0%; 5-yr OS 88.7%		
							OS, 1.6 (1.2-2.3)	12-24 wks	112	5-yr EFS 68.6%; 5-yr OS 78.4%		
Nurgalieva et al. [20] 2013	USA (Retros., BCCA)	100% pts ≥65 yr	Post.	I-III	NR	Polychemotherapy	Median FU: NR	Total	14,380		8	Age, marriage status, tumor stage, size, grade, hormone receptor status, comorbidity, year of diagnosis, SEER region, primary surgery and radiotherapy, chemotherapy, and race/ethnicity
							Reference	≤3 months	12,748			
							OS, 1.53 (1.32–1.80)	>3 months	1,632			
							DSS, 1.83 (1.31–2.47)	>3 months	1,632			

Abbreviations: *Anthr*, Anthracycline; *BCCA*, British Columbia Cancer Agency; *CI*, Confidence interval; *CMF*, Cyclophosphamide, methotrexate, and fluorouracil; *CT*, Clinical trial; *DBCG*, Danish Breast Cancer Cooperative Group; *DFS*, Disease-free survival; *DSS*, Disease-specific survival; *EFS*, Event-free survival; *FASG*, French Adjuvant Study Group; *FU*, Follow up; *HR*, Hazard ratio; *IBCSG*, International Breast Cancer Study Group; *LN+*, Lymph nodes positive; *NR*, Not reported; *OS*, Overall survival; *Post*, Postmenopausal; *Pre*, Premenopausal; *Pros*, Prospective study; *Retro*, Retrospective study; *RFS*, Relapse-free survival; *SEER*, The Surveillance, Epidemiology, and End-Results database; *WT*, Waiting time.

^*^The publish type of this study is a meeting abstract.

^§^Analysis performed in patients receiving chemotherapy only.

^**^Evaluated by the 9-star Newcastle-Ottawa Scale.

**Figure 2:**
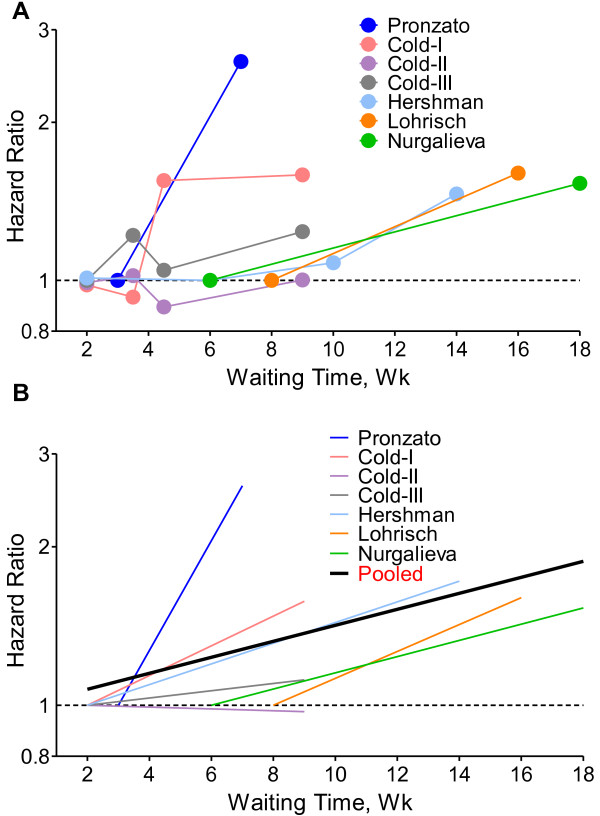
**Individual hazard ratio for overall survival according to waiting time categories. A**. The relationship between waiting time categories and overall survival in the 7 independent analytical groups. The hazard ratio (HR) represents a comparison with the lowest waiting time category in each study (as reference). The first author of each study is shown. **B**. Conversion of HR estimates from the original studies to an HR per week of delay. The slope of each line represents the change in the log HR per week delay. The line for each individual study is located over the range of waiting times. The thick line indicates the weighted average of the HRs from the individual studies. The vertical axis is on a log scale.

The HR results from individual eligible studies listed in Table 1 are plotted in Figure 2A, which shows the HRs for categorical representations of waiting time in the 7 studies for OS. The waiting times covered by the studies ranged from 2 to 12 weeks. This figure illustrates that HRs at different waiting time were similar and therefore supports conversion of HRs from categories to an HR for a continuous representation by waiting time. For each study, a single HR corresponding to the relative increase in mortality risk with each additional 4-week of waiting time was extracted (Figure 2B). For studies contrasting 2 waiting time categories, the line was the same as that presented in Figure 2A. For studies using more than 2 categories, the HR was estimated using meta-regression. The 4-folds of slope of each line (by log converted HR) in Figure 2B represented the log of final HR used in meta-analysis (i.e., HR per 4-week of delay).

**Figure 3:**
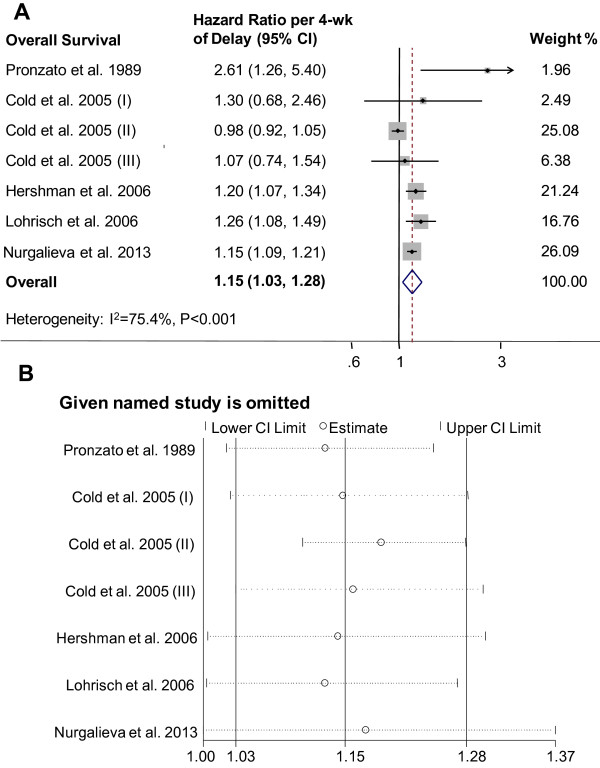
**Individual study and overall hazard ratios of relationships between every 4-week delay in initiation of adjuvant chemotherapy and overall survival.** Individual and overall hazard ratios (HR) per 4-week of delay with 95% confidence interval (CI) for OS are shown in **A**. The size of each square is proportional to the weight of the study. For the combined result, the length of the diamond represents the 95% CI of the summary. **B**. shows the influence of individual studies on the pooled HR. The vertical axis indicates the overall HR and the two vertical axes indicate its 95% CI. Every hollow round indicates the pooled OR when the left study is omitted in this meta-analysis. The two ends of every broken line represent the respective 95% CI.

Figure 3A presents the forest plot of meta-analysis for OS, including HRs and 95% CIs per 4-week of delay for 7 analytical groups. The combined HR was 1.15 (95% CI, 1.03-1.28; P = 0.009) by random-effects model. There was statistically significant heterogeneity between studies of OS (P < 0.001; I^2^ = 75.4%). To explore the resource of heterogeneity, we performed influence analysis, which omits one study at a time and calculates the recombined HRs for the remainders. It showed that the Cold-II study by Cold et al. [10] substantially influenced the pooled HR (Figure 3B). After excluding that single study, there was no between-study heterogeneity (P = 0.257; I^2^ = 23.6%), and the HR was more significant (HR = 1.17; 95% CI, 1.12-1.22; P < 0.001; fixed-effects model). To further test the robustness of our study, we alternatively removed 2 studies with the largest weight and recalculated a combined HR estimate from the remaining studies, consistent and statistically significant results were maintained. The HR after removal of the Cold-II study by Cold et al. [10] (25.08% weight) and the study by Nurgalieva et al. [20] (26.09% weight) was 1.23 (95% CI, 1.12-1.34; fixed-effects model) without evident heterogeneity either (P = 0.284, I^2^ = 20.5%). The funnel plot was used to evaluate publication bias and the Egger’s test showed no evidence of publication bias (P = 0.351).

**Figure 4:**
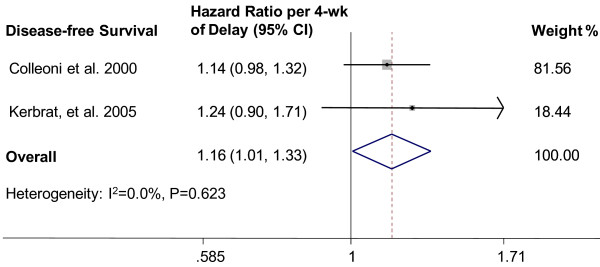
**Individual study and overall hazard ratios of relationships between every 4-week delay in initiation of adjuvant chemotherapy and disease-free survival.** Individual and overall hazard ratios (HR) per 4-week of delay with 95% confidence interval (CI) for DFS is shown. The size of each square is proportional to the weight of the study. For the combined result, the length of the diamond represents the 95% CI of the summary.

The analyses were repeated for DFS (forest plot shown in Figure 4). The relevant 2 studies included 4,390 breast cancer patients. The combined HR was 1.16 (95% CI, 1.01-1.33; fixed-effects model), without evidence of heterogeneity (P = 0.623, I^2^ = 0.0%).

## Discussion

Adjuvant chemotherapy (AC) has been admitted as the standard treatment for most breast cancer patients. However, the exact time frame of AC treatment initiated post-surgery to gain maximal benefit still remains unclear. The published clinical trials do not specifically suggest the timing of chemotherapy after surgery, and there is a wide variation across trials in the allowed time between surgery and AC, ranging from 2 to 12 weeks [21-24]. It is unlikely that there will be additional prospective clinical trials comparing outcomes for AC initiation before or after a specified time (not perioperative) from surgery. Therefore, we have to rely on retrospective data as reviewed in this study. In this report, the systematic review and meta-analysis indicate that OS decreases by 15% for every 4-week delay in initiation of AC. Our results are also consistent across DFS analysis. This present study is the first fully-reported meta-analysis specifically addressing the effect of a delay in time to AC on survival outcomes in breast cancer in a quantitative way.

The effect of AC on survival is thought to be eradication of micrometastatic deposits in a proportion of patients. There is a substantial theoretical rationale to initiate AC immediately after curative surgery. Investigation in animal models has demonstrated that surgery may increase the numbers of circulating tumor cells and oncogenic growth factors, and accelerate growth of metastases [25,26]; a single dose of chemotherapy given early seemed more efficient than treatment given later [27]. Biological plausibility, clinical observations, and published studies have brought up a comprehensive hypothesis that early initiation of AC is clinically crucial to patient’s survival.

The available evidence that describes a relationship between time to AC and patient outcomes is shown in Table 1. In other relevant studies of association between time to AC and survival but not included in this meta-analysis due to low validity, inconsistent results were presented. Studies by Buzdar et al. [28], Shannon et al. [29], Samur et al. [4], and Sanchez et al. [30] failed to show inferior outcome for chemotherapy started later after surgery compared with chemotherapy stared early. In contrast, Alkis et al. [31] reported that OS was significantly better in patients who started AC within 44 days. Brooks et al. [32] also exhibited an improvement in DFS for patients with node-positive cancers receiving AC within 4 weeks compared with those patients receiving delayed chemotherapy. Another Turkish study [33] argued that the upper limit of time to initiation of AC could be up to 4.8 months. We did not included all the aforementioned studies [4,28-33] since none of them have provided sufficient data to calculate an adjusted and quantitive HR for meta-analysis. Biagi et al. [34] also performed a similar meta-analysis and demonstrated that a 4-week increase in time to adjuvant chemotherapy was associated with a significant OS HR of 1.06 (95% CI 1.02-1.10) and DFS HR of 1.08 (95% CI 1.03-1.14) in breast cancer. However, that study published abstract only in ASCO 2011, and there was a statistical flaw because the authors combined individual studies using a fixed-effect model although there was an obvious inter-study heterogeneity.

Our meta-analysis demonstrates an evident association between delayed AC and poor OS. However, there was a significant heterogeneity between studies for OS. By influence analysis, a study (Cold-II) based on clinical trial data [10] seemed to be a major resource of heterogeneity. After removing that single study, the heterogeneity disappeared and the association was more significant. The disparate results before and after removing the Cold-II study [10] may be due to the relative short waiting times of that study (all the patients from controlled trials and received AC within 3-months after surgery), patient selection bias (women with delayed AC could not be enrolled in original trials), inappropriate category classification (investigator grouped the patients into 1–3 weeks, 4 weeks, 5 weeks, and 6–13 weeks group; such short intervals make detection of significance difficult), and possibly, the cycle numbers of chemotherapy (they used CMF i.v. on day 1, every 3 weeks, 9 times; while classical CMF used on days 1 and 8, every 4 weeks, 12 times).

Applying our findings to a patient who is ready to initiate AC at 4 weeks after surgery but is actually delayed, this patient would have a 15% increased risk of mortality if treated at 8 weeks and 32.25% increased risk at 12 weeks. According to the updated EBCTCG report, 36% reduction in breast cancer mortality rate can be achieved for AC versus no AC at 10-year [1]. We may reckon that, in general, breast cancer patients should start AC with no more than 8-week delay of the planned initiation which is probably within 4 weeks after surgery. However, for the high-risk patients with young age and ER-negative tumor, individualized strategy of AC initiation should be applied according to relevant study [9]. Although our analysis may over- or under-estimate the effect of delayed time on survival, we believe these results should help to modify protocols for those agencies that carry breast cancer cares and services.

Some limitations should be declared. First, our meta-analysis is limited by the nonrandomized and retrospective nature of the included studies. However, it is unrealistic to expect that a randomized trial of time to AC will ever be done; rather, analyses such as ours are likely to provide the only evidence of such an effect. Hence we believe that our results, coupled with preclinical models and relevant clinical evidence, have provided sufficient proof of a substantial reverse relationship between prolonged waiting times to initiation of AC and reduced survival. Second, there should be other prognostic factors not controlled in the meta-analysis. The number of cycles, completion rate for AC, dose reduction, using endocrine therapy or not, and HER2 status, which were considered as key determinants of survival, are not always adjusted in the eligible studies. The effect of AC delay on survival might vary in patients with different clinicopathological features. However, because of a lack of individual information of patients, we failed to do sub-analyses according to different features. Third, at least 57% of all the study patients (according to Hershman’s [6] and Nurgaliev’s [20] studies) were older than 65 years. The different age distribution of the patients between this study and general breast cancer population (median age is 55 years according to SEER database [35]) might have potential impacts on the conclusion. Fourth, our study relies on the assumption of a log-linear relationship for the effect of waiting time on survival. However, the assumption of linearity to this relationship might be problematic sometimes. For instance, a few studies showed that survivals were similar for patients if they started AC within 12 weeks after surgery, and only those starting AC at later than 12 weeks had significantly inferior survival [6,7]. Since a linear relationship may unfit the first 12 weeks, the regressed summary HR across the whole time frame may not reflect the real effect. Finally, since most included studies used CMF and anthracycline-based regimen, whether the results of meta-analysis can be extrapolated to the current taxane era is unclear. Albeit this, our findings might potentially have broad clinical relevance. Since removal of a primary tumor would enhance the growth of metastasis [26,36], it is plausible that early intervention of conventional cytotoxic agents (anthracycline, cyclophosphamide, methotrexate, etc.) would exert a better tumor suppressive effect. Comparing with classic cytotoxic agents, taxanes are more effective on cells in division and growth since they are microtubule inhibitors that bind reversibly to the subunit of tubulin and lead to cell arrest at the G2/M phase of the cell cycle. It is reasonable to speculate that early initiation of taxane-containing chemotherapy may be particularly effective on inhibiting the cancer cells in mitotic phase caused by surgery stress.

## Conclusion

Our results demonstrate a significant adverse association between waiting time to AC initiation and survival in breast cancer. The results also provide further validation of the intuitive concept of early time to AC after surgical treatment. Physicians may need to give more careful consideration to timing when discussing AC with patients, and clinicians and jurisdictions need to optimize the patient flow logistics to minimize the interval from surgery to AC.

## Competing interest

The authors have declared that no competing interests exist.

## Authors’ contributions

YKD, HS, ZJX, LGY and SZM drafted the manuscript. YKD, HS, and ZJX participated in data collection and analysis. YKD, HS, and SZM participated in data interpretation. YKD and SZM participated in the conception and design of the study. YKD designed the general study. All authors reviewed and approved the final manuscript.

## Pre-publication history

The pre-publication history for this paper can be accessed here:

http://www.biomedcentral.com/1471-2407/13/240/prepub
